# Cenerimod, a selective S1P_1_ receptor modulator, improves organ-specific disease outcomes in animal models of Sjögren’s syndrome

**DOI:** 10.1186/s13075-021-02673-x

**Published:** 2021-11-29

**Authors:** Estelle Gerossier, Saba Nayar, Sylvie Froidevaux, Charlotte G. Smith, Celine Runser, Valentina Iannizzotto, Enrico Vezzali, Gabin Pierlot, Ulrich Mentzel, Mark J. Murphy, Marianne M. Martinic, Francesca Barone

**Affiliations:** 1 Idorsia Pharmaceuticals Ltd., Hegenheimermattweg 91, 4123 Allschwil, Switzerland; 2 Centre for Translational Inflammation Research, Institute of Inflammation and Ageing, College of Medical & Dental Sciences, University of Birmingham Research Laboratories, Queen Elizabeth Hospital, Birmingham, UK; 3 Rheumatology Department, University Hospitals Birmingham NHS Foundation Trust, Birmingham, UK

**Keywords:** Sjögren’s syndrome, Sphingosine-1-phosphate receptor type 1, Animal models, Immunomodulation, Cenerimod

## Abstract

**Background:**

Sjögren’s syndrome is a systemic autoimmune disease characterized by immune cells predominantly infiltrating the exocrine glands and frequently forming ectopic lymphoid structures. These structures drive a local functional immune response culminating in autoantibody production and tissue damage, associated with severe dryness of mucosal surfaces and salivary gland hypofunction. Cenerimod, a potent, selective and orally active sphingosine-1-phosphate receptor 1 modulator, inhibits the egress of lymphocytes into the circulation. Based on the mechanism of action of cenerimod, its efficacy was evaluated in two mouse models of Sjögren’s syndrome.

**Methods:**

Cenerimod was administered in two established models of Sjögren’s syndrome; firstly, in an inducible acute viral sialadenitis model in C57BL/6 mice, and, secondly, in the spontaneous chronic sialadenitis MRL/lpr mouse model. The effects of cenerimod treatment were then evaluated by flow cytometry, immunohistochemistry, histopathology and immunoassays. Comparisons between groups were made using a Mann-Whitney test.

**Results:**

In the viral sialadenitis model, cenerimod treatment reduced salivary gland immune infiltrates, leading to the disaggregation of ectopic lymphoid structures, reduced salivary gland inflammation and preserved organ function. In the MRL/lpr mouse model, cenerimod treatment decreased salivary gland inflammation and reduced T cells and proliferating plasma cells within salivary gland ectopic lymphoid structures, resulting in diminished disease-relevant autoantibodies within the salivary glands.

**Conclusions:**

Taken together, these results suggest that cenerimod can reduce the overall autoimmune response and improve clinical parameters in the salivary glands in models of Sjögren’s syndrome and consequently may reduce histological and clinical parameters associated with the disease in patients.

**Supplementary Information:**

The online version contains supplementary material available at 10.1186/s13075-021-02673-x.

## Background

Sjögren’s syndrome is a systemic autoimmune disease that predominantly affects the exocrine glands, mainly the salivary and lacrimal glands, and leads to severe dryness of mucosal surfaces and salivary gland hypofunction. The aetiology of Sjögren’s syndrome is controversial and most likely multifactorial. Affected glands are massively infiltrated by lymphocytes, predominantly CD4^+^ T cells, but also CD8^+^ T cells [1] and B cells [2]. Although T cells prevail in mild lesions, B cells are the most represented lymphocytes in the advanced lesions [3]. In 30–40% of patients, infiltrating lymphocytes are organized into clusters or ectopic lymphoid structures (ELS) that recapitulate histological and functional features of active follicles present within lymph nodes, including the presence of activated T cells and proliferating B cells, and plasma cells associated with follicular dendritic cells [4]. Presence of ELS in Sjögren’s syndrome has been included as part of the diagnostic criteria and is expressed as a quantification of aggregates over 4 mm^2^ area (focus score) [5, 6]. ELS formation is not specific to Sjögren’s syndrome but is also associated with other autoimmune diseases and chronic inflammation as observed in rheumatoid arthritis and autoimmune thyroiditis [7, 8]. ELS appear to play a significant role in sustaining disease progression in autoimmune conditions [9, 10]. Despite significant improvements in disease understanding and management, Sjögren’s syndrome largely remains an indication of high unmet medical need [11].

Migration of activated lymphocytes across different anatomical barriers is largely responsible for driving disease in different organs and is an attractive therapeutic target in autoimmune conditions [12]. The sphingosine-1-phosphate (S1P) receptor type 1 (S1P_1_) plays a major role in the migration of lymphocytes via interaction with its ligand S1P [13–19]. High S1P concentrations in the vascular and lymphatic circulation and lower concentrations in thymus and lymph nodes generate a gradient, promoting egress of lymphocytes out of peripheral lymphoid organs into the circulation via the S1P_1_ receptor [20]. Accordingly, pharmacological modulation of S1P_1_ leads to sequestration of T and B lymphocytes in lymphoid organs. S1P receptor modulators are approved to treat multiple sclerosis, providing evidence that this mode of action, the inhibition of migration of lymphocytes into the circulation and target tissues, is of importance in complex autoimmune conditions [21].

In patients with Sjögren’s syndrome, the S1P/S1P_1_ axis has been shown to contribute to disease pathogenesis through increased expression of S1P_1_ in salivary gland mononuclear cells and increased S1P-driven IFN-γ production by CD4^+^ T cells, resulting in enhanced IFN-γ-mediated Fas expression and Fas-dependent apoptosis of salivary gland epithelial cells [22]. Based on the central role of S1P_1_ in lymphocyte trafficking and its contribution to Sjögren’s syndrome pathogenesis, modulation of S1P_1_ was assessed in ameliorating Sjögren’s syndrome disease. Specifically, cenerimod, a potent, selective and orally active S1P_1_ receptor modulator [23], currently being tested in a multi-dose safety and efficacy Phase 2b study in patients with systemic lupus erythematosus (SLE) (NCT03742037), was evaluated in two mouse models reflecting some biological and clinical features of Sjögren’s syndrome. Firstly, an inducible model of sialadenitis was utilized in which sialadenitis is induced through the administration of a replication-defective type-5 adenovirus (AdV5) in the submandibular gland of wild-type C57BL/6 mice. AdV5-cannulated mice develop several features of Sjögren’s syndrome, such as formation of ELS within the salivary glands, ectopic expression of lymphoid chemokines, development of humoral autoimmunity to nuclear antigens and a decrease in salivary flow [24]. Secondly, the spontaneous sialadenitis MRL/lpr mouse model was utilized. This model is a well-recognized chronic model of Sjögren’s syndrome that spontaneously develops in an age-dependent manner and reflects key features of autoimmune sialadenitis in conjunction with lupus-like symptoms [25]. With increasing age, MRL/lpr mice demonstrate decreased salivary flow rate [26–28] and a decrease in tear production [29]. Furthermore, destructive lymphocytic infiltrates in the salivary [28] and lacrimal glands are observed [28], and anti-Ro/SSA and anti-La/SSB autoantibodies are present [30]. Thus, these models offer a valuable possibility to dissect the cellular and molecular mechanisms regulating breach of tolerance, autoimmunity and ELS formation in the salivary glands.

## Methods

### Animals

#### C57BL/6 viral sialadenitis model

Viral sialadenitis was performed as previously described [24]. Briefly, 12-week-old female C57BL/6 mice (Charles River) were cannulated with 1e8-1e9 pfu replication-deficient AdV5 into the salivary gland on day 0. All mice were maintained under specific pathogen-free conditions in the BMSU.

#### MRL/lpr lupus model

Four-week-old female MRL/MpJ-Fas^lpr^/J mice (Jackson Laboratories) were group-housed under climate-controlled conditions with a 12-h light / dark cycle, a standard temperature of 20 ± 3 °C and appropriate environmental enrichment (cardboard, tissues, red polycarbonate houses and seeds) in the cages. Mice had free access to food and drinking water ad libitum.

### Study procedure

#### C57BL/6 viral sialadenitis model

In the early and late therapeutic regimen, cenerimod was administered at 10 mg/kg once daily (q.d.) per os (p.o.), 2 or 8 days following cannulation with AdV5, respectively, until the end of the study (day 15). Each therapeutic arm had matched vehicle (0.5% methylcellulose containing 0.5% Tween® 80 in water)-treated mice.

On day 15, for saliva samples, mice were given general anaesthesia and were then secured in the supine position. Salivation was induced by subcutaneous administration of 10 mg/kg pilocarpine (Sigma-Aldrich) in phosphate-buffered saline (PBS). Saliva was collected with a pipet over a 10-min period and transferred into weighed Eppendorf tubes, the tubes were then weighed a second time and the volume of saliva calculated (1 mg = 1 μL saliva). Results were expressed as μL saliva / 10 min/g body weight.

All mice were then euthanized with terminal anaesthesia (EUTHASOL®). Blood was collected into EDTA tubes via vena cava puncture. Submandibular salivary gland and draining cervical lymph nodes were removed, weighed and processed accordingly for enzymatic digestion, histology or cytokine profiling.

#### MRL/lpr lupus model

Cenerimod was prepared as a mix in the regular food pellet (Granovit AG) at a final concentration of 200 mg/kg food. At this concentration and based on a daily food intake of 2–4 g per animal, the mice would receive a daily dose of 20–40 mg/kg/day. The food pellet alone was used as the control treatment (vehicle).

At 6 weeks of age, after the acclimatization period, mice were randomly assigned to the vehicle or cenerimod group (*n* = 20 per group) based on body weight (week 0) and started receiving vehicle or cenerimod treatment one week later (week 1). Treatment continued until the end of the study, which was predefined as the time point when at least 20% morbidity/mortality was reached in one group.

At treatment week 11 (17 weeks of age), all mice were euthanized with an overdose of intraperitoneal 150 mg/kg injection of pentobarbital natricum (Streuli Pharma SA). Blood was collected into EDTA tubes via vena cava puncture and plasma prepared with centrifugation for 5 min at 14,000 rpm at 4 °C (Eppendorf Centrifuge 5417R). Animals were then perfused with PBS at 37 °C and necropsied. Salivary glands were collected and weighed.

### Immunophenotyping

#### C57BL/6 viral sialadenitis model

Peripheral blood (50 μL) and salivary glands from cenerimod-treated and vehicle-treated mice were taken from culled animals at day 15 post cannulation. Salivary glands were dissected and placed in 1 mL of RPMI-1640 (with 2% foetal calf serum (FCS)) on ice. For isolation of single cells, the tissues were digested in 2 mL enzyme mix (RPMI-1640 with 2% FCS, 0.8 mg/mL dispase, 0.2 mg/mL collagenase P and 0.1 mg/mL DNase I) as previously described [31].

Suspensions were incubated with a purified anti-mouse CD16/32 antibody (Fc Block, 2.4G2, BD Biosciences) for 10 min followed by staining for 30 min with cocktail of the following antibodies: CD45 (30-F11), CD3e (145–2 C11), CD4 (RM4-5), CD19 (1D3) (all from eBiosciences), CD8a (53-6.7) and CD138 (281-2) (BD biosciences). After the staining, erythrocytes from blood were lysed using RBC Lysis buffer (Sigma) according to the manufacturer’s instructions.

The cells were washed, and single-cell suspensions were reconstituted in PBS with 0.5% BSA and 2 mM EDTA. Cells were stained with fixable viability dye (eBiosciences) and fixed with 4% PFA before acquisition to exclude dead cells as per manufacturer’s instructions. Cells were analysed using Fortessa (BD) with forward/side scatter gates set to exclude non-viable cells. Data were analysed with FlowJo software (Tree Star).

#### MRL/lpr lupus model

Leukocytes were collected from peripheral blood (50 μL) and half of the left salivary gland. Salivary gland suspension was prepared by enzymatic digestion for 15 min at 37 °C in RPMI-1640 medium (Gibco), DNase I 20 U/mL (Invitrogen) and Liberase DL 500 μg/mL (Roche).

Suspensions were incubated with a purified anti-mouse CD16/32 antibody (Fc Block, 2.4G2, BD Biosciences) for 10 min followed by staining for 30 min with an antibody cocktail specific for the different leukocyte subsets including B cells and T cells. The following antibody conjugates were purchased from Biolegend: PB anti-mouse CD45 (30-F11), BV510 anti-mouse CD69 (H1.2F3), PE anti-mouse CD317 (PDCA) (129C1), PE-Cy7 anti-mouse β TCR (H57-597), AF700 anti-mouse CD19 (6D5) and APC-Cy7 anti-mouse CD4 (GK1.5). The following antibodies were purchased from BD Bioscience: AF488 anti-mouse CD45R/B220 (RA3-6B2), PerCP-Cy5.5 anti-mouse CD8a (53-6.7), PB anti-mouse CD4 (RM4-5).

After the staining, erythrocytes from blood were lysed using RBC Lysis buffer (Biolegend) according to the manufacturer’s instructions.

The cells were washed, and single-cell suspensions were reconstituted in PBS, 0.5% BSA, 0.03% NaN3 and 2 mM EDTA. Propidium iodide (Calbiochem) was added before the acquisition to identify dead cells. The cells were acquired on a Gallios Cytometer (Beckman Coulter) and absolute number determined using Trucount Tubes (BD Bioscience).

Analysis was performed using Kaluza analysis 1.5a software. For analysis, the cells were gated in forward scatter versus side scatter, followed by a doublet exclusion. A live gate was performed for all samples. In the blood, the B cell population was defined as CD19^+^ B220^+^. T cells were defined as TCRβ^+^ CD19^−^ and the T cell subpopulations were further defined as CD4^+^ CD8^−^ (CD4^+^ T cells), CD4^−^ CD8^+^ (CD8^+^ T cells) and CD4^−^ CD8^−^ (double-negative T cells). In the salivary gland, the B cell population was defined as CD45^+^ B220^+^ TCRβ^−^ CD317^−^. T cells were defined as CD45^+^ TCRβ^+^ and the T cell subpopulations were further defined as CD4^+^ CD8^−^ (CD4^+^ T cells), CD4^−^ CD8^+^ (CD8^+^ T cells) and CD4^−^ CD8^−^ (double-negative T cells). The activation of B and T cells were evaluated using a CD69^+^ stain.

### Cytokine measurements

#### C57BL/6 viral sialadenitis model and MRL/lpr lupus model

After sampling and weighing, the left and right salivary glands (C57BL/6 viral sialadenitis model) or half of the left salivary gland (MRL/lpr lupus model) were placed into 1 mL Tris 50 mM / NaCl 0.1 M / Triton 0.1% buffer containing 1% protease inhibitor cocktail (Sigma Aldrich) in a lysing Matrix D tube (MP Biomedicals). Samples were then homogenized on a FastPrep 24 5G (MP Biomedicals) and centrifuged for 10 min at 14,000 rpm at 4 °C (Eppendorf Centrifuge 5417R). Supernatants were used for cytokine/chemokine determination. A Milliplex Mouse Cytokine/Chemokine Magnetic kit (Millipore) was used to quantify the cytokine/chemokine concentration in salivary gland supernatants following the manufacturer’s protocol. Samples were run undiluted on a Luminex 200 system using Luminex xPONENt software. Results are expressed as picograms per salivary gland (mean of right and left salivary gland; C57BL/6 viral sialadenitis model) or as picograms normalized to the whole left salivary gland (MRL/lpr lupus model).

### Immunohistochemistry (IHC)

#### C57BL/6 viral sialadenitis model

Immunofluorescence (IF) staining was performed on salivary glands and cervical lymph nodes obtained from AdV5-infected mice treated with cenerimod or vehicle as previously described [32]. The following antibodies were used: mouse CD45 (30-F11), CD19 (eBio1D3) and CD3e (ebio500A2) (all from eBiosciences).

In order to assess the number and dimension of foci (lymphocytic aggregates: > 50 cells) as well as the degree of lymphoid organization in cannulated salivary glands from cenerimod or vehicle-treated mice, CD3 and CD19 were utilized for identifying T cells and B cells, respectively. CD3/CD19 double-stained slides were acquired on a Zeiss 880 Confocal Microscope and tile scans for the whole salivary gland section were taken. A region around the foci was drawn on images in the Zen 2012 (blue edition) software, then both the area of the foci and the area for the whole tissue section was determined using the software. This numerical data was used to calculate the:

- Focus score = number of foci per 4 mm^2^ of tissue area
- Average foci area = total foci area/number of foci
- Area fraction = total foci area/total area

Based on CD3/CD19 double staining, we also counted foci that were segregated (i.e. aggregates in which T and B cells are organized in separate areas) as an indicator of lymphoid organization).

- Percentage of segregation = (number of segregated foci/total number of foci) × 100

All measurements were performed in a masked fashion by observers in a blinded manner.

#### MRL/lpr lupus model

After sampling, the right salivary gland was fixed in 10% neutral buffered formalin (4% formaldehyde) (J.T. Baker Inc.) at room temperature for 24 h, and paraffin embedded. Hematoxylin and eosin-stained paraffin sections of 2 μm were assessed by bright field microscopy. For immunohistochemistry analysis, paraffin sections were processed to 4-μm sections. The staining approach consisted of serial applications of Tyramide Signal Amplification (TSA)-amplified immunofluorescence labels (PerkinElmer) on the Leica Bond RX (Leica Biosystems). Briefly, slides were deparaffinized using Bond Dewax solution RX (Leica Biosystems) on the Leica Bond-RX autostainer; epitope retrieval was performed with HIER (Heat-Induced Epitope Retrieval) (citrate-based pH 6.0 solution; Leica Biosystems). After blocking endogenous peroxidase (Leica Biosystems), staining was performed using primary antibodies against CD3 (SP7, Abcam), CD19 (EPR23174-145, Abcam), CD138 (281-2, BD Biosciences) and Ki67 (SP6, Genetex) followed by an HRP-conjugated secondary antibody (Jackson ImmunoResearch). Slides were counterstained with DAPI (Merck) and mounted with BrightMount/Plus Mounting Medium (Abcam). FFPE sections without primary antibodies were used as negative control in each batch of IHC staining. Multiplex IHC using TSA were applied for immune cell population characterization in ectopic lymphoid structures. Three fluorophores were used: TSA Plus Cyanine 3.5, fluorescein and Cyanine 5.5 (Akoya Biosciences).

Fluorescence images were acquired (× 40) using the NanoZoomer S60 Digital slide scanner (Hamamatsu). The data from the multispectral camera were accessed by Orbit Imaging software (http://www.orbit.bio). Ectopic lymphoid structures were delineated manually as region of interest. Following the annotation, immune cell populations were characterized and quantified using a classification model.

### Quantitative RT-PCR

#### C57BL/6 viral sialadenitis model

Total RNA was isolated from thick cryosections of murine salivary glands and cervical lymph nodes with an RNeasy mini kit (Qiagen). RNA was then reverse transcribed using the high capacity reverse transcription cDNA synthesis kit (Applied Biosystems) according to the manufacturer’s specifications. Reverse transcription was carried out on an Eppendorf Thermal Cycler PCR machine. Quantitative real-time (qRT)-PCR (Applied Biosystems) was performed on cDNA samples for *ccl19*, *cxcl13*, *lta*, *ltb* and *aicda* mRNA expression. β-actin was used as an endogenous control. The primers and probes used were from Applied Biosystems. qRT-PCR was run in duplicates on a 384-well PCR plate (Applied Biosystems) and detected using an ABI PRISM 7900HT instrument. Results were analysed with the Applied Biosystems SDS software (SDS V.2.3) as previously described [32].

### BAFF, AID and CXCL13 concentrations

#### C57BL/6 viral sialadenitis model and MRL/lpr lupus model

BAFF concentrations in salivary gland homogenates were measured by quantitative ELISA using the Mouse BAFF/BLyS/TNFSF13B kit (R&D Systems) according to the manufacturer’s protocol. Salivary gland homogenates were used undiluted. The absorption was measured at 450 nm using a Spectromax 384 microplate reader (Spectromax Molecular Devices). For the C57BL/6 viral sialadenitis model, results are expressed as nanograms per salivary gland (mean of right and left salivary gland). For the MRL/lpr lupus model, results are expressed as nanograms normalized to the whole left salivary gland.

#### MRL/lpr lupus model

AID (activation-induced cytidine deaminase) concentrations in salivary gland homogenates were measured by Competitive Elisa kit (MyBioSource) according to the manufacturer’s protocol. Salivary gland homogenates were used undiluted. The absorption was measured at 450 nm using a Spectromax 384 microplate reader (Spectromax Molecular Devices). Results are expressed as picorgrams normalized to the whole left salivary gland.

CXCL13 concentrations in salivary gland homogenates were measured using a U-plex kit (Mesoscale Discovery) according to the manufacturer’s protocol. Salivary gland homogenates were used diluted at 1:4 in diluent buffer. The electrochemiluminescence signal was measured using the MSD Sector Imager S-600 reader and analysed with SoftmaxPro software (Molecular Device). Results are expressed as picograms normalized to the whole left salivary gland.

### Anti-SSB antibody titres

#### MRL/lpr lupus model

Anti-SSB (La) antibody titres were measured by ELISA using the Mouse Anti-SSB (La) kit (Creadive Diagnostics) according to the manufacturer’s protocol. Plasma samples were diluted in diluent buffer at 1:100, and salivary gland homogenates were used diluted at 1:2. The absorption was measured at 450 nm using a Spectromax 384 microplate reader (Spectromax Molecular Devices, USA). Results are expressed as optical density after subtraction of the blank value (dilution buffer). They were normalized to the whole left salivary gland.

### Anti-dsDNA antibody titres

#### MRL/lpr lupus model

Anti-double-stranded DNA (dsDNA) antibody titres were measured by ELISA using a modified version of a previously described method [33].

Briefly, calf thymus DNA sodium salt (Sigma Aldrich) was used to coat 96-well plates overnight at 20 μg/mL at 4°C and then blocked with /PBS / 2% BSA (KPL) for 1 h at 37 °C. After washing, samples were added; plasma samples were diluted 1:800 in PBS / 1% BSA / 0,2% Tween, and salivary gland homogenates were left undiluted. After 1 h incubation at 37 °C, anti-mouse polyvalent immunoglobulins (IgG, IgM)–alkaline phosphatase antibody (Sigma Aldrich) was added for 2 h at 37 °C, and the reaction developed with Alkaline Phosphate Yellow Liquid substrate (Sigma Aldrich) for 1 h at 37 °C. The absorption was read at 405 nm using a Spectromax 384 microplate reader (Spectromax Molecular Devices). Results are expressed as optical density after subtraction of the blank value (dilution buffer). They were normalized to the whole left salivary gland.

### Serum anti-AdV5 IgG ELISA titres

#### C57BL/6 viral sialadenitis model

Relative virus-specific serum antibody titres were evaluated by ELISA. Peripheral blood obtained from mice was allowed to clot at room temperature and serum was separated from corpuscular component by spinning at 13,000 rpm for 10 min. Sera were aliquoted and frozen at − 20°C until further use. To measure for viral-specific antibody titre, the AdV solution (that is used to cannulate mice) was used to coat ELISA plates (MaxiSorp plates, Nunc) at 5 μg/mL in coating buffer (1 capsule of Sigma carbonate-bicarbonate buffer / 100 mL H_2_O). After an overnight incubation at 4 °C, plates were washed three times with PBS and blocked with 100 μL of PBS / 1% BSA for 1 h at 37 °C, to prevent non-specific binding in subsequent steps. Post incubation, plates were washed again three times with PBS. Sera were diluted 1:20 in PBS / 1% BSA / 0.05% Tween 20 and added to the first row of the 96-well ELISA plate. For each of the samples, further fourfold serial dilutions in PBS / 1% BSA / 0.05% Tween 20 were prepared in each column and then the plate was incubated for 2 h at 37 °C followed by three times washing with PBS. Afterwards, in each well, 200 μL of serum diluted (1:20) with PBS (2 replicates for each sample) was added. Serial dilutions were then made for each sample. The plate was incubated with the sera overnight at 4 °C. After incubation, 100 μL of anti-mouse IgG-HRP (fc specified pre-absorbed) diluted 1:2000 in PBS / 1% BSA / 0.05% Tween 20 as detecting antibodies was added, after washing, for each well. To detect the signal, after washing, 70 μL of 1-Step Ultra TMB-ELISA Substrate (Thermo Fisher) was added to each well, and the reaction was stopped after 10 min with the addition of 70 μL HCl 0.5M. Optical density values at 405 nm were obtained using the SoftmaxPro software (Molecular Device). Relative antibody titres for each sample were obtained by plotting the dilution against the optical density values.

### Statistical analyses

GraphPad Prism Software version 7.04 for Windows was used for all data analysis and graphical illustrations. Comparisons between groups were made using a Mann-Whitney test; *p* values < 0.05 were considered statistically significant.

Heatmap profiles were performed using R version 4.0.3. R (Foundation for Statistical Computing). Heatmaps show cytokine concentration levels. Row *Z* scores are used to improve rendering; in addition, rows are reordered based on the fold change between vehicle and cenerimod groups. An agglomerative hierarchical clustering defined using Euclidean distance and Ward’s linkage criterion was used within each group to reorder animals.

## Results

### Cenerimod treatment reduces blood and salivary gland tissue infiltrates in the viral sialadenitis mouse model

**Fig. 1:**
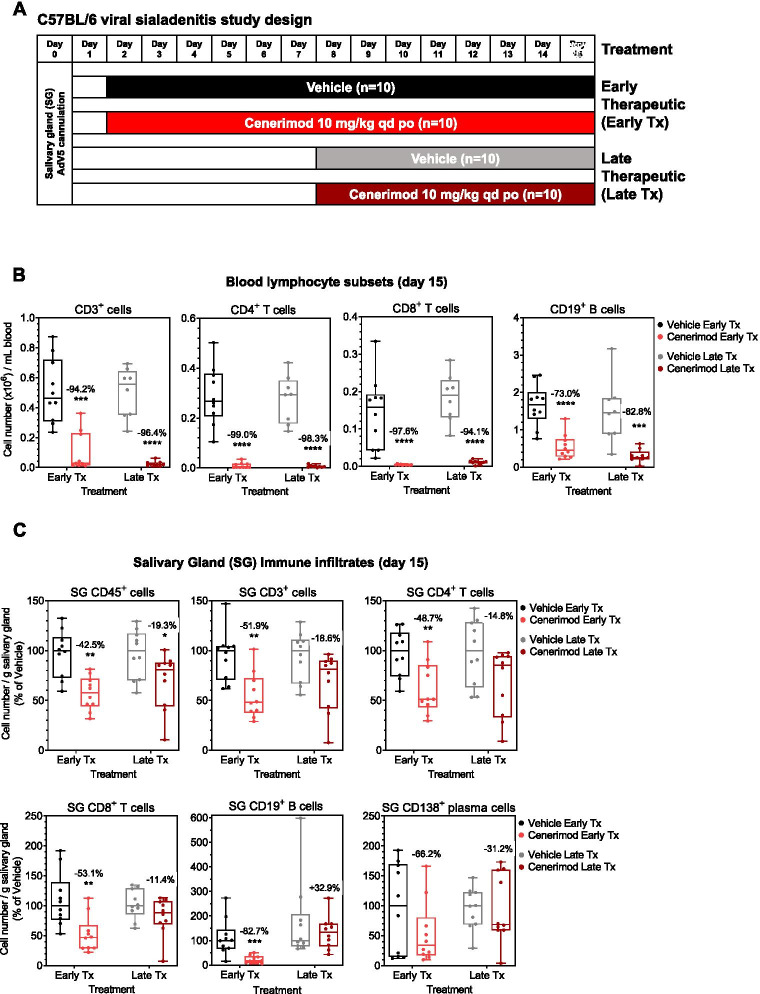
Cenerimod treatment reduces blood and salivary gland tissue infiltrates in the viral sialadenitis mouse model. **A** Viral sialadenitis study design in C57BL/6 female mice depicting early and late therapeutic regimens; mice were 12 weeks of age at the time of AdV5 cannulation (day 0). **B** Blood lymphocyte subsets were quantified at the end of treatment (day 15) by flow cytometry in cenerimod- and vehicle-treated animals. Numbers in the graphs indicate the percent change versus vehicle. **C** Quantification of salivary gland lymphocytic subset infiltrates at the end of treatment (day 15) by flow cytometry. Cell numbers were normalized to gram salivary gland and shown as a percentage of vehicle. Numbers in the graphs indicate the percent change versus vehicle. **B,C** Each data point represents the measurement of individual mice from three independent experiments with 3–4 mice per group; horizontal line indicates the median, the box indicates the upper and lower quartiles and the whiskers indicate the minimum and maximum range; *p < 0.05, **p < 0.01, ***p < 0.001, ****p < 0.0001 vs. vehicle group (Mann-Whitney test). Tx, therapeutic; SG, salivary gland; qd, once daily; po, per os

In order to evaluate the beneficial impact of cenerimod treatment in ameliorating aspects of Sjögren’s syndrome disease, cenerimod was tested in a virus-induced sialadenitis model [24]. Mice were treated with cenerimod in two therapeutic regimens to mimic the effect of S1P_1_ receptor blockade in the early or established phase of the disease (Fig. 1A). Cenerimod treatment, in both regimens, resulted in significantly reduced blood lymphocyte counts with a reduction in both CD4^+^ and CD8^+^ T cells and B cell populations compared with vehicle controls (Fig. 1B) and in accordance with its published mode of action [34]. The reduction in circulating lymphocytes had no impact on the ability of cenerimod-treated mice to mount a pathogen-specific immune response as shown by the presence of anti-AdV5 IgG titres comparable to vehicle-treated animals at the end of the study (Additional file 1).

Reflecting the effect elicited by cenerimod treatment on peripheral lymphocytes, salivary gland CD45^+^ immune cell infiltrates were significantly reduced in both treatment regimens as compared to vehicle control (Fig. 1C). In the late therapeutic treatment mode, the reduction in CD4^+^ and CD8^+^ T cells, and B cells, was less pronounced than observed in the early therapeutic regimen, where these reductions were significant as compared to vehicle controls. In addition, in both cenerimod treatment groups, a numerical decrease in plasma cells was observed (Fig. 1C).

### Cenerimod treatment modulates salivary gland pathology in the viral sialadenitis mouse model

**Fig. 2:**
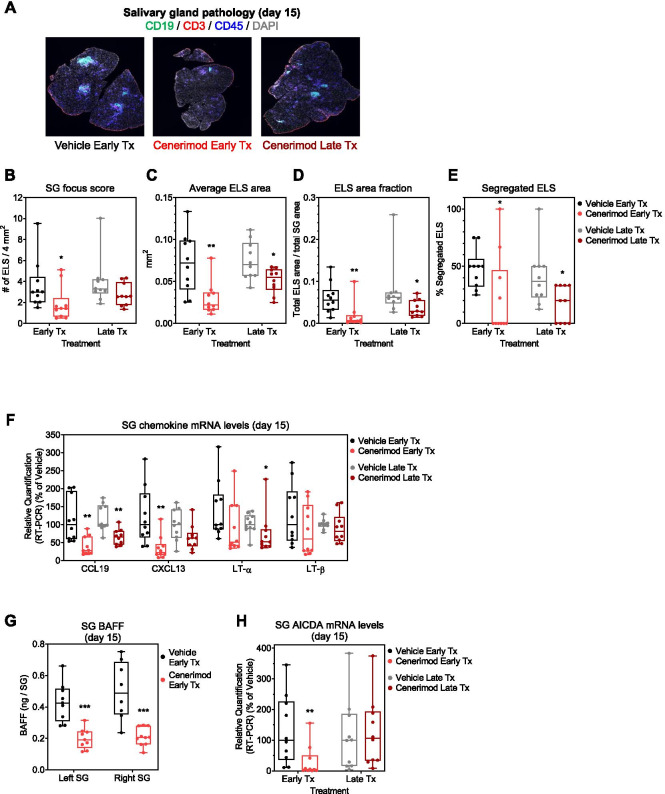
Cenerimod treatment modulates salivary gland pathology in the viral sialadenitis mouse model. **A** Representative microphotographs of salivary glands at the end of the study (day 15), depicting CD19^+^ B cells (green), CD3^+^ T cells (red), CD45^+^ leukocytes (blue) and cell nuclear counterstain (DAPI, grey), as shown by immunofluorescence. **B** Salivary gland focus score as measured by numbers of ELS per 4 mm^2^. **C** Average ELS area measurement in mm^2^. **D** ELS area fraction as measured by the total ELS area divided by the total salivary gland area. **E** Percentage of segregated ELS. **F** Salivary gland chemokine mRNA levels (CCL19, CXCL13, LT-α, LT-β) at the end of treatment (day 15) measured by quantitative real-time PCR; cenerimod groups are shown as a percentage of vehicle. **G** Salivary gland B cell activating factor (BAFF) protein levels were measured at the end of early therapeutic treatment (day 15) using a quantitative ELISA and shown in ng/SG. **H** Salivary gland chemokine activation-induced cytidine deaminase (AICDA) mRNA levels at the end of treatment (day 15) measured by quantitative real-time PCR; cenerimod groups are shown as a percentage of vehicle. **B–H** Each data point represents the measurement of individual mice from three independent experiments with 3–4 mice per group; horizontal line indicates the median, the box indicates the upper and lower quartiles and the whiskers indicate the minimum and maximum range; *p < 0.05, **p < 0.01, ***p < 0.001 vs. vehicle group (Mann-Whitney test). Tx, therapeutic; SG, salivary gland; ELS, ectopic lymphoid structures; LT, lymphotoxin

Immunohistochemistry revealed that cenerimod treatment led to a reduction in salivary gland histopathology as depicted by a reduction in both the size and number of ELS within the salivary gland (Fig. 2A). Specifically, cenerimod treatment reduced the degree of immune cell infiltration as demonstrated by a reduced focus score (Fig. 2B), decreased average ELS area (Fig. 2C), smaller ELS area fraction (Fig. 2D), and a reduction in ELS organization (Fig. 2E).

The reduction in ELS was reflected in an overall decrease in chemokines required for ELS assembly and maintenance within the tissue in both treatment regimens, specifically CCL19, CXCL13, LT-α and LT-β [9] (Fig. 2F). In addition, there was a significant reduction in factors associated with autoantibody promotion within the salivary gland in cenerimod-treated animals. Specifically, B cell activating factor (BAFF) (Fig. 2G) and the gene expression for activation-induced cytidine deaminase (AICDA) were significantly lower in the salivary glands of cenerimod-treated animals in the early therapeutic group (Fig. 2H).

Even though lymphocytes were significantly decreased in the blood of cenerimod- as compared to vehicle-treated mice in both treatment regimens (Fig. 1B), cenerimod treatment did not disrupt secondary lymphoid organization (Additional file 1) nor affect the production of lymphoid chemokines in the draining lymph node (Additional file 1).

### Cenerimod treatment modulates the salivary gland inflammatory environment and improves saliva production in the viral sialadenitis mouse model

**Fig. 3:**
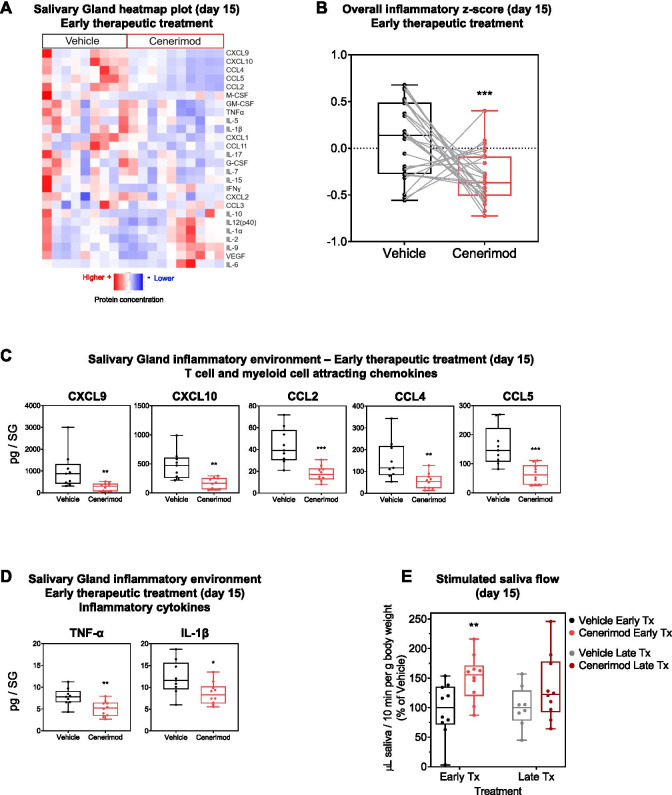
Cenerimod treatment reduces local inflammation and improves salivary gland function in the viral sialadenitis model. **A** Heatmap plots from 26 cytokine/chemokines using Luminex technology with red representing high protein concentrations and blue low protein concentrations. Each vertical line represents protein values from one individual mouse taken at the end of early therapeutic treatment (day 15). **B** Overall inflammatory score represented as box and whiskers plot. Data points represent, for each cytokine, the median within each group after scaling and centering the values. A line is drawn between two points to join the values of each cytokine in both groups. **C,D** Quantification of protein concentrations of T cell and myeloid cell attracting chemokines (**C**) and inflammatory cytokines (**D**) from the salivary gland using Luminex technology at the end of treatment (day 15). **E** Saliva production as measured by microlitres of saliva produced in 10 min per gram body weight at the end of treatment (day 15). Cenerimod groups are shown as a percentage of vehicle. **C–E** Each data point represents the measurement of individual mice from three independent experiments with 3–4 mice per group; horizontal line indicates the median, the box indicates the upper and lower quartiles and the whiskers indicate the minimum and maximum range; *p < 0.05, **p < 0.01, ***p < 0.001 vs. vehicle group (Mann-Whitney test); Tx, therapeutic; SG, salivary gland

Cenerimod treatment led to a significant reduction in a number of inflammatory cytokines within the salivary gland (Fig. 3A and Additional file 1). The reduction in the inflammatory environment within the salivary gland was also apparent by a decrease in the overall normalized *z*-score of all the cytokines measured (Fig. 3B). In particular, T cell and myeloid cell attracting chemokines such as CXCL9 and CXCL10, CCL2, CCL4 and CCL5 (Fig. 3C), as well as the inflammatory cytokines TNF-α and IL-1β (Fig. 3D) were significantly reduced.

The reduction in the overall inflammatory environment within the salivary gland was reflected in an improved salivary gland function, measured by stimulated saliva flow. Cenerimod treatment led to an improved saliva production as compared to vehicle controls in both therapeutic regimens but this was more pronounced in the early therapeutic regimen (Fig. 3E).

### Cenerimod treatment reduces blood and salivary gland infiltrates in the MRL/lpr mouse model

**Fig. 4:**
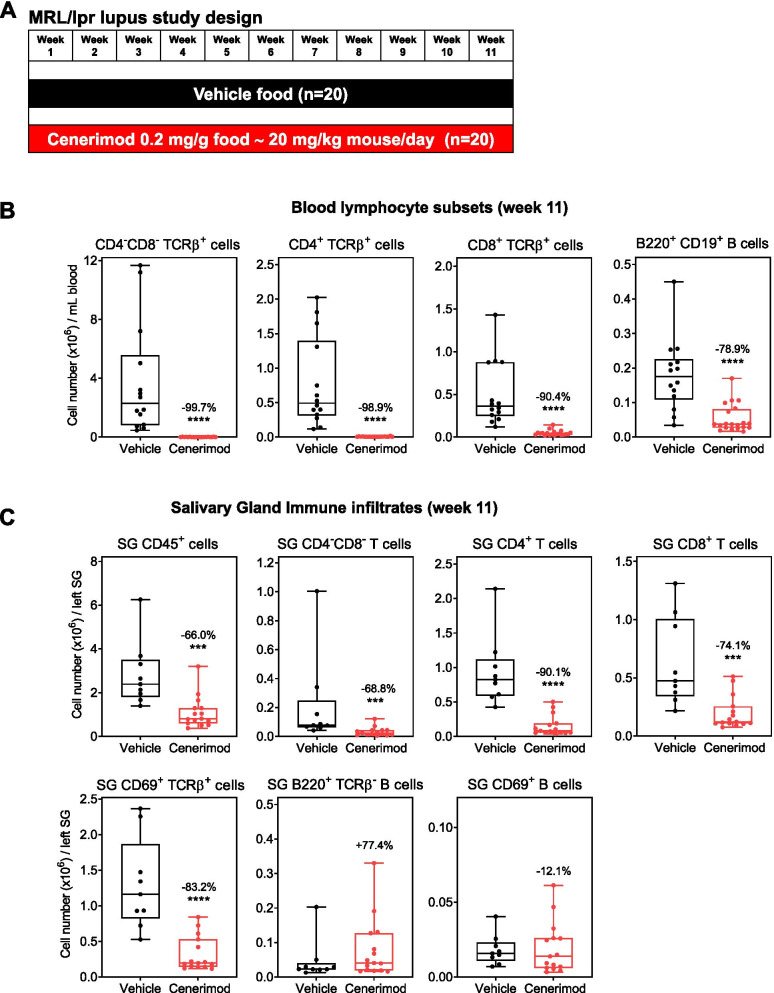
Cenerimod treatment reduces blood and salivary gland infiltrates in the MRL/lpr mouse model. **A** Study design in MRL/lpr female mice; mice (*n* = 20 per group) were 7 weeks of age at treatment start (week 1). Study was pre-defined to end when 20% morbidity/mortality was reached in one group, which was reached at the end of week 10, and all mice were sacrificed at the beginning of week 11. **B** Blood lymphocyte subsets were quantified at the end of treatment (week 11) by flow cytometry in cenerimod- and vehicle-treated animals. Numbers in the graphs indicate the percent change versus vehicle. **C** Quantification of salivary gland lymphocytic subset infiltrates at the end of treatment (week 11) by flow cytometry. Cell numbers were normalized to the whole left salivary gland. Numbers in the graphs indicate the percent change versus vehicle. **B,C** Each data point represents the measurement of individual mice; horizontal line indicates the median, the box indicates the upper and lower quartiles and the whiskers indicate the minimum and maximum range; ***p < 0.001, ****p < 0.0001 vs. vehicle group (Mann-Whitney test). SG, salivary gland

In order to assess the effect of cenerimod treatment in a chronic model that also reflects features of clinical Sjögren’s syndrome, the MRL/lpr mouse model was chosen. In this model, mice spontaneously develop, in an age-dependent manner, autoimmune sialadenitis in conjunction with lupus-like symptoms [25]. At 7 weeks of age, an age at which B cell abnormalities are already detected [35], MRL/lpr mice were treated with cenerimod for 10 weeks, at which point the pre-defined endpoint of at least 20% mortality in one group was reached (6/20 vehicle-treated animals vs. 0/20 cenerimod-treated animals) [34] (Fig. 4A). As has been previously described in this model [34], cenerimod treatment led to a significant blood lymphocyte reduction (Fig. 4B). Importantly, CD45^+^ immune cell infiltrates within the salivary gland were significantly reduced in cenerimod-treated animals at the end of the study as compared to vehicle control (Fig. 4C). Specifically, CD4^−^CD8^−^, CD4^+^ and CD8^+^ T cells, including activated CD69^+^ T cells, were significantly reduced (Fig. 4C). The total B cell population was not significantly elevated in salivary glands of cenerimod-treated animals, and the number of activated CD69^+^ B cells remained unchanged (Fig. 4C).

### Cenerimod treatment decreases the local inflammatory environment within the salivary gland in the MRL/lpr mouse model

**Fig. 5:**
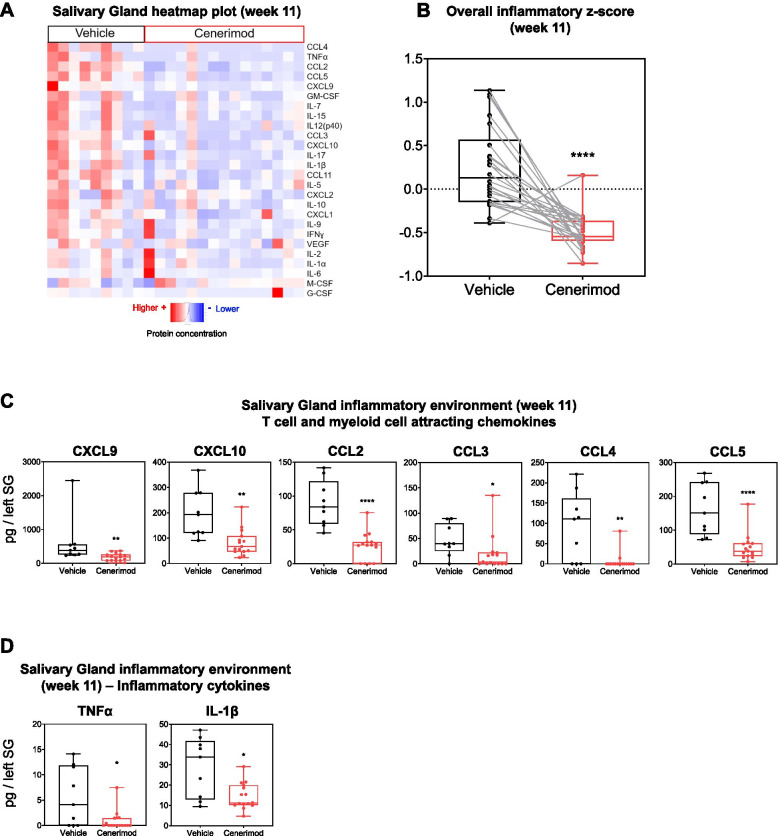
Cenerimod treatment decreases the local inflammatory environment within the salivary gland in the MRL/lpr model. **A** Heatmap plots from 26 cytokine/chemokines using Luminex technology with red representing high protein concentrations and blue low protein concentrations. Each vertical line represents protein values from one individual mouse taken at the end of the treatment (week 11). **B** Overall inflammatory score represented as box and whiskers plot. Data points represent, for each cytokine, the median within each group after scaling and centering the values. A line is drawn between two points to join the values of each cytokine in both groups. **C,D** Quantification of protein concentrations of T cell and myeloid cell attracting chemokines (**C**) and inflammatory cytokines (**D**) from the salivary gland using Luminex technology. Each data point represents the measurement of individual mice at the end of the treatment (week 11); horizontal line indicates the median, the box indicates the upper and lower quartiles and the whiskers indicate the minimum and maximum range; *p < 0.05, **p < 0.01, ****p < 0.0001 vs. vehicle group (Mann-Whitney test); SG, salivary gland

Consistent with the viral sialadenitis model, cenerimod treatment led to a pronounced reduction of the inflammatory environment within the salivary gland (Fig. 5A and Additional file 1). This was also reflected in an overall reduction of the normalized inflammatory *z*-score (Fig. 5B). Particularly, the IFN-associated chemokines CXCL9 and CXCL10, and the T cell and myeloid cell attracting chemokines CCL2, CCL3, CCL4 and CCL5 were significantly reduced (Fig. 5C). In addition, the pro-inflammatory cytokines TNF-α and IL-1β were significantly reduced (Fig. 5D) as were Th1/Th17-specific cytokines (IFN-γ, IL-17, GM-CSF; Additional file 1).

### Cenerimod treatment reduces T cells and proliferating plasma cells within the salivary gland ELS in the MRL/lpr mouse model

**Fig. 6:**
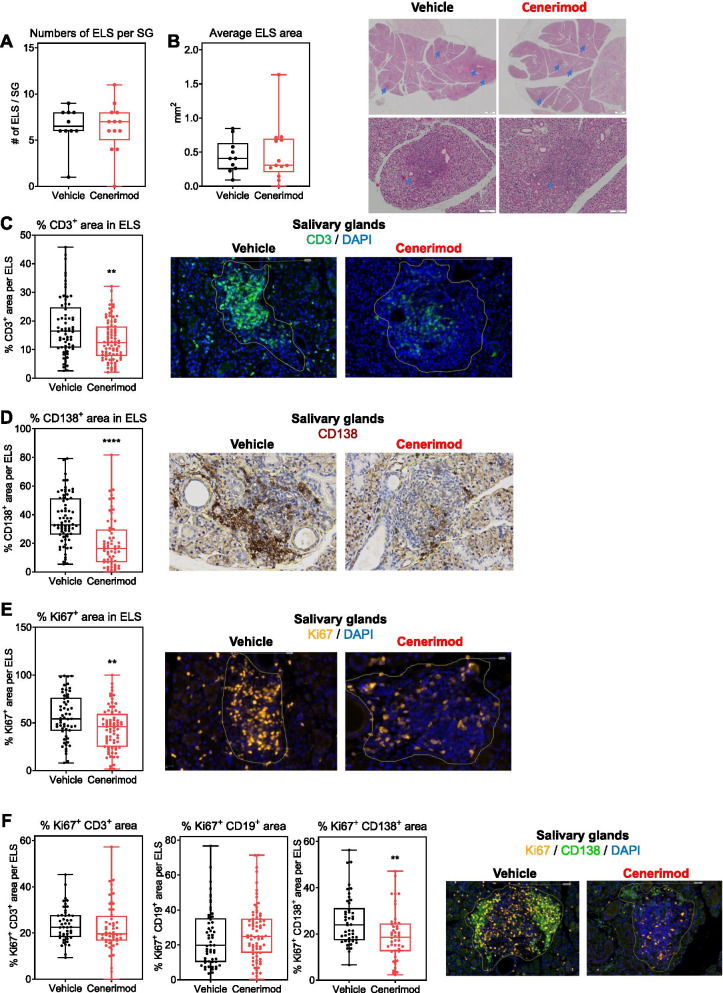
Cenerimod treatment reduces T and proliferating plasma cells within the ELS in the MRL/lpr model. Quantification of the salivary gland ELS (**A**), the average ELS area (in mm^2^) (**B**) and representative H&E microphotographs of mandibular salivary glands (**B**); blue arrows indicate ELS. **C** Quantification of CD3^+^ T cell area as a percentage of ELS area; representative microphotographs of salivary glands depicting CD3^+^ T cells (green) and cell nuclear counterstain (DAPI, blue); the yellow line marks the ELS border. **D** Quantification of CD138^+^ cell area as a percentage of ELS area; representative microphotographs of salivary glands depicting CD138^+^ plasma cells (dark brown) and cell nuclear counterstain (blue); the yellow line marks the ELS border. **E** Quantification of Ki67^+^ cell area as a percentage of ELS area; representative microphotographs of salivary glands depicting Ki67^+^ cells (orange) and cell nuclear counterstain (DAPI, blue); the yellow line marks the ELS border. **F** Quantification of Ki67^+^ CD3^+^, Ki67^+^ CD19^+^ and Ki67^+^ CD138^+^ area as a percentage of ELS area; representative microphotographs of salivary glands depicting Ki67^+^ cells (orange), CD138^+^ plasma cells (green), Ki67^+^ CD138^+^ cells (yellow) and cell nuclear counterstain (DAPI, blue); the yellow line marks the ELS border. In all graphs, each data point represents the measurement of individual mice at the end of the study (week 11); horizontal line indicates the median, the box indicates the upper and lower quartiles and the whiskers indicate the minimum and maximum range; **p < 0.01, ****p < 0.0001 vs. vehicle group (Mann-Whitney test). SG, salivary gland; ELS, ectopic lymphoid structures

Histological analysis of the salivary glands of MRL/lpr animals at the time of sacrifice identified consistently ELS in vehicle-treated animals confirming previous observations [25]. Macroscopically, both the number and the average area of the ELS were comparable between cenerimod-treated and vehicle controls (Fig. 6A, B). However, within ELS, the T cell population was significantly reduced in cenerimod-treated animals (Fig. 6C). Importantly, immunohistochemistry analysis also revealed that plasma cells within ELS were significantly reduced by cenerimod treatment (Fig. 6D). To confirm that these CD138^+^ plasma cells were bona fide CD138^+^CD3^−^ plasma cells and not aberrant CD138^+^CD3^+^ cells as shown to have been detected in MRL/lpr mice [36], salivary glands were stained with CD138- and CD3-specific antibodies. The staining confirmed that the majority of CD138^+^ cells were indeed bona fide plasma cells (Additional file 1). Moreover, a significant reduction in Ki67 staining, a marker of proliferation, was observed in ELS of cenerimod-treated animals as compared to vehicle (Fig. 6E). Strikingly, the reduction in Ki67 staining was specific to plasma cells as both the proliferating Ki67^+^CD3^+^ T cell and Ki67^+^CD19^+^ B cell populations within ELS remained unchanged between the two groups (Fig. 6F).

### Cenerimod treatment reduces autoantibodies within the salivary gland in the MRL/lpr mouse model

**Fig. 7:**
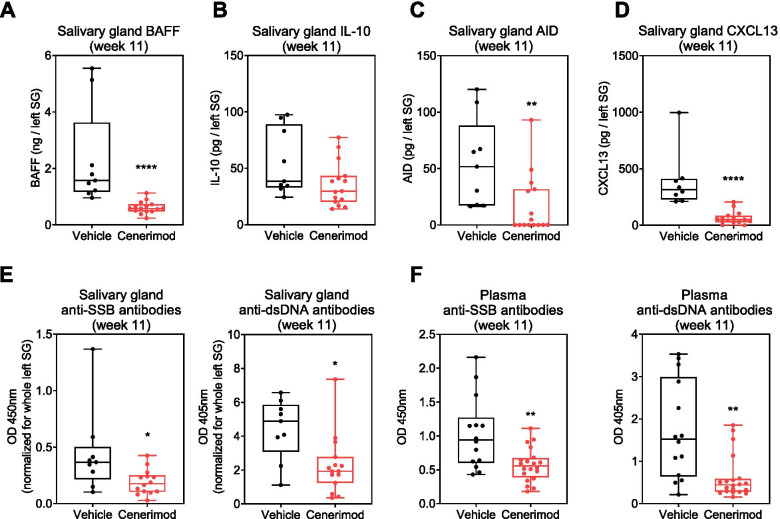
Cenerimod treatment reduces autoantibodies within the salivary gland in the MRL/lpr mouse model. Quantification of salivary gland B cell activating factor (BAFF) protein (ng/SG) (**A**), IL-10 protein (pg/SG) (**B**), activation-induced cytidine deaminase (AID) protein (pg/SG) (**C**) and CXCL13 protein (pg/SG) (**D**). Quantification of salivary gland (**E**) and plasma (**F**) anti-SSB (OD 450 nm) and anti-dsDNA antibodies (OD 405 nm). Each data point represents the measurement of individual animals at the end of the study (week 11); horizontal line indicates the median, the box indicates the upper and lower quartiles and the whiskers indicate the minimum and maximum range; *p < 0.05, **p < 0.01, ****p < 0.0001 vs. vehicle group (Mann-Whitney test). SG, salivary gland; OD, optical density

The reduction in T cells and plasma cells within the ELS was also associated with a reduction in factors shown to promote autoantibody production (Fig. 7A–D), with similarities to what was observed in the viral sialadenitis model (Fig. 2). Specifically, BAFF, IL-10 and AID protein levels were reduced (Fig. 7A–C). Furthermore, the B cell recruiting factor CXCL13 was also significantly reduced (Fig. 7D).

Importantly, the reduction in T cells and plasma cells together with a decrease in factors known to promote autoantibody production, also correlated with overall less productive ELS as measured by a significant reduction of autoantibodies associated with Sjögren’s syndrome. Specifically, cenerimod treatment led to a significant decrease in both anti-SSB and anti-dsDNA antibodies in the salivary glands (Fig. 7E); a significant reduction was also observed in the plasma (Fig. 7F).

## Discussion

Despite significant improvements in disease understanding and management, achieving disease remission in Sjögren’s syndrome, and preserving organ function is still considered challenging [11].

In the current study, we evaluated the effect of cenerimod, a selective S1P_1_ receptor modulator in two mouse models of Sjögren’s syndrome, the acute induced viral sialadenitis model and the spontaneously developing chronic MRL/lpr model [24, 25]. Consistent with its activity in sequestering immune cells in secondary lymphoid organs, cenerimod was able to decrease the number of circulating and salivary gland-infiltrating immune cells in both disease models. This effect was reflected in reduced productive ELS associated with diminished autoantibodies and preserved organ function.

Importantly, the sequestering of lymphocytes by cenerimod had no impact on the ability of the immune system to mount a specific antibody response to an acute infection of adenovirus serotype 5 used in the viral sialadenitis model and provides evidence that the selective effects of cenerimod on lymphocyte subsets allows key immune responses to be preserved which has also been shown previously with other S1P_1_ receptor modulators [37].

Within the salivary glands of patients with Sjögren’s syndrome, infiltrating leukocytes together with stromal cells frequently form organized aggregates, often referred to as ELS [38], which promote antigen-specific adaptive immune responses that exacerbate chronic disease. The generation and maintenance of these structures are governed by the precise interplay of lymphocytes and stromal cells and defined inflammatory signals [39]. In the acute viral sialadenitis model, the reduction in activated infiltrating T cells in cenerimod-treated animals perturbed the ability to form ELS as demonstrated in both numerical reduction and decrease in organizational structure of the ELS, highlighting the importance of T cells in this process. Furthermore, in cenerimod-treated salivary glands, chemokines and cytokines central to the assembly and maintenance of ELS [40], in particular CCL19, CXCL13, LT-α and LT-β were reduced, further underlining the importance of T cell/ectopic lymphoid tissue crosstalk. This was only apparent in ELS as importantly, the organization of secondary lymphoid organs remained unaffected following cenerimod treatment. In the chronic MRL/lpr model, although the ELS were not reduced in number in cenerimod-treated animals compared to vehicle control, the reduction in infiltrating activated T cells within the ELS was significant. The reduction in T cells in both models were associated with functional consequences, i.e. improved saliva production in the former and a decrease in local autoantibodies in the latter. Interestingly, FTY720, a non-selective S1P receptor modulator was also able to reduce immune cell infiltrates into salivary glands in the spontaneous NOD-AEC1.AEC.2 mouse model of Sjögren’s syndrome which also led to improved salivary gland function [41]. Together with our findings, these data suggest that the impact on improved salivary gland function is due to the modulation of S1P_1_ and not other S1P receptors. Modulation of the S1P_1_ receptor pathway was previously also shown to disrupt productive ELS in a hypersensitive pneumonitis model [42] suggesting an indirect role of S1P_1_ receptor signaling in ELS biology in other diseases as well.

The reduction of infiltrating lymphocytes driven by cenerimod treatment observed in both models correlated with a significant reduction in BAFF, a critical survival factor for mature B cells and promoter of B cell proliferation, differentiation and IgG class switching [43]. Furthermore, CXCL13 and IL-10, which are both associated with an autoantibody promoting environment [44–46], were also reduced. The importance of BAFF in Sjögren’s syndrome was recently demonstrated using a monoclonal therapy targeting BAFF in a recent phase 2 clinical trial which resulted in a reduction in disease activity [47]. In addition, CXCL13 strongly correlates with local pathology in Sjögren’s syndrome patients [48] and elevated IL-10 levels in plasma and saliva correlate with disease severity and autoantibody titres [44–46]. Further, AID, an enzyme expressed in activated B cells and essential for promoting somatic hypermutation and class switch recombination, and increased in patients with Sjögren’s syndrome [49], was reduced in both animal models after treatment, suggesting a diminished functional B cell response. Supporting this observation, within the ELS of cenerimod-treated MRL/lpr salivary glands, there was a significant reduction in proliferating plasma cells. Autoantibody-producing plasma cells are increased in the salivary glands of Sjögrens syndrome patients and numerous studies have implicated plasma cells in driving pathology in Sjögren’s syndrome [50]. The reduction of proliferating plasma cells suggests a loss in productivity of the ELS which is further strengthened by the observation that the anti-dsDNA and anti-SSB autoantibodies, a hallmark of Sjögren’s syndrome [51], were reduced in the salivary glands and in the plasma of cenerimod-treated MRL/lpr animals.

ELS formation is associated with a chronic local inflammatory salivary gland environment which is detrimental to tissue function in Sjögren’s syndrome patients [38, 52]. The inflammatory environment is recapitulated to a strong degree both in the viral sialadenitis and the MRL/lpr model. Specifically, the IFN-γ-responsive chemokines CXCL9 and CXCL10, and the T cell and myeloid chemoattractants CCL2, CCL3, CCL4 and CCL5, and the pro-inflammatory cytokines TNF-α and IL-1β, all of which are elevated in patients and correlate with more active disease [53–60], were decreased in cenerimod-treated animals. What remains to be shown clinically, is whether decreasing T cell infiltrates in the salivary gland has potentially wider ranging consequences in also modulating chemokines associated with an innate immune response, known to be dysregulated in Sjögren’s disease [61].

Interestingly, in Sjögren’s syndrome the relationship between inflammation and organ damage is not linear, suggesting that additional factors, besides cell infiltration and damage, contribute to loss of glandular function [62, 63]. Nonetheless, in our study, an amelioration in glandular function was observed, including an increase in saliva production, in the viral infected mice treated with cenerimod compared to vehicle control. Improved efficacy observed in the early therapeutic vs. late therapeutic regimen may reflect the time required to remove lymphocytes from both the circulation and tissue and reduce the associated damage before a significant improvement can be observed. Additionally, cenerimod treatment in the MRL/lpr mice commenced at 7 weeks of age at a time point when lymphocyte infiltration into the salivary gland is apparent but has not fully developed [64]. These results suggest that early treatment may provide a window of opportunity to treat Sjögren’s syndrome patients before glandular function is compromised irreversibly.

Taken together, these results suggest that cenerimod can reduce the overall auto-immune response and improve clinical parameters in the salivary glands in models of Sjögren’s syndrome and consequently may reduce histological and clinical parameters associated with the disease in patients. Interestingly, in a phase 2 study in patients with SLE, an autoimmune disorder with multiple circulating autoantibodies and often associated with Sjögren’s syndrome [65], cenerimod was able to significantly decrease anti-dsDNA antibody titres, prevent an increase of systemic IFN-α and reduce the number of circulating antibody-secreting cells already after 12 weeks of treatment [34, 66]. These encouraging data suggest that cenerimod may be a promising approach to treating Sjögren’s syndrome.

## Conclusions

By sequestering both T and B lymphocytes and consequently reducing salivary gland infiltrates, our data provide evidence that cenerimod has the capacity of targeting multiple disease-associated mechanisms in animal models of Sjögren’s syndrome, providing encouraging evidence that cenerimod may also be effective in treating Sjögren’s syndrome in humans.

## Supplementary Information

**Additional file 1: Supplementary figure 1**. Serum anti-AdV5 IgG ELISA titres in vehicle and early therapeutic cenerimod treated animals at the end of the study (day 15). Results represented as mean ± SEM of three independent experiments with 3-4 mice per group. AdV5, adenovirus type 5; Tx, therapeutic; SEM, standard error of the mean. **Supplementary figure 2**. (A) Representative microphotographs of draining cervical lymph nodes at the end of the study (day 15), depicting CD3^+^ T cells (fuchsia), CD19^+^ B cells (green), and CD45^+^ leukocytes (blue) of vehicle and early therapeutic cenerimod treated animals, as shown by immunofluorescence. (B) Cervical lymph node chemokine mRNA levels (CCL19, CXCL13, LT-a, LT-β) in vehicle and early therapeutic cenerimod treated animals at the end of the study (day 15) measured by quantitative real-time PCR; cenerimod groups are shown as a percentage of vehicle. Each data point represents the measurement of individual mice from three independent experiments with 2-3 mice per group; horizontal line indicates the median, the box indicates the upper and lower quartiles, and the whiskers indicate the minimum and maximum range (Mann-Whitney test). LT, lymphotoxin; Tx, therapeutic. **Supplementary figure 3**. Box plots of cytokine and chemokine proteins measured in vehicle and early therapeutic cenerimod treated C57BL/6 mice at the end of the study (day 15). Each data point represents the measurement of individual mice from three independent experiments with 2-4 mice per group; horizontal line indicates the median, the box indicates the upper and lower quartiles, and the whiskers indicate the minimum and maximum range. *p<0.05, **p<0.01, ***p<0.001 vs. vehicle group (Mann-Whitney test). SG, salivary gland. **Supplementary figure 4**. Box plots of cytokine and chemokine proteins measured in vehicle and cenerimod treated MRL/lpr mice at the end of the study (week 11). Each data point represents the measurement of individual mice; horizontal line indicates the median, the box indicates the upper and lower quartiles, and the whiskers indicate the minimum and maximum range. *p<0.05, **p<0.01, ***p<0.001, ****p<0.0001 vs. vehicle group (Mann-Whitney test). SG, salivary gland. **Supplementary figure 5**. Representative microphotographs of salivary glands of vehicle- (left column) or cenerimod-treated (right column) MRL/lpr mice at the end of the study (week 11). The microphotographs depict CD138^+^ cells (red) and CD3^+^ T cells (green), as shown by immunofluorescence.

## Data Availability

Upon reasonable request.

## Abbreviations

AdV5: Adenovirus type 5
AICDA, AID: Activation-induced cytidine deaminase
BAFF: B cell activating factor
ELS: Ectopic lymphoid structures
LT: Lymphotoxin
S1P: Sphingosine-1-phosphate
S1P_1_: Sphingosine-1-phosphate receptor type 1
SG: Salivary gland
SLE: Systemic lupus erythematosus
TMB: Tetramethylbenzidine
