# Supplementary material for: Cenerimod, a selective S1P1 receptor modulator, improves organ-specific disease outcomes in animal models of Sjögren’s syndrome
Source: Arthritis Res Ther. 2021 Nov 29;23:289. doi: 10.1186/s13075-021-02673-x (PMC8628476; doi:10.1186/s13075-021-02673-x)
Supplement: Supplementary file 1 — Additional file 1: Supplementary figure 1. Serum anti-AdV5 IgG ELISA titres in vehicle and early therapeutic cenerimod treated animals at the end of the study (day 15). Results represented as mean ± SEM of three independent experiments with 3-4 mice per group. AdV5, adenovirus type 5; Tx, therapeutic; SEM, standard error of the mean. Supplementary figure 2. (A) Representative microphotographs of draining cervical lymph nodes at the end of the study (day 15), depicting CD3+ T cells (fuchsia), CD19+ B cells (green), and CD45+ leukocytes (blue) of vehicle and early therapeutic cenerimod treated animals, as shown by immunofluorescence. (B) Cervical lymph node chemokine mRNA levels (CCL19, CXCL13, LT-a, LT-β) in vehicle and early therapeutic cenerimod treated animals at the end of the study (day 15) measured by quantitative real-time PCR; cenerimod groups are shown as a percentage of vehicle. Each data point represents the measurement of individual mice from three independent experiments with 2-3 mice per group; horizontal line indicates the median, the box indicates the upper and lower quartiles, and the whiskers indicate the minimum and maximum range (Mann-Whitney test). LT, lymphotoxin; Tx, therapeutic. Supplementary figure 3. Box plots of cytokine and chemokine proteins measured in vehicle and early therapeutic cenerimod treated C57BL/6 mice at the end of the study (day 15). Each data point represents the measurement of individual mice from three independent experiments with 2-4 mice per group; horizontal line indicates the median, the box indicates the upper and lower quartiles, and the whiskers indicate the minimum and maximum range. *p<0.05, **p<0.01, ***p<0.001 vs. vehicle group (Mann-Whitney test). SG, salivary gland. Supplementary figure 4. Box plots of cytokine and chemokine proteins measured in vehicle and cenerimod treated MRL/lpr mice at the end of the study (week 11). Each data point represents the measurement of individual mice; horizontal line indicates [file 13075_2021_2673_MOESM1_ESM.pptx]

## Slide 1
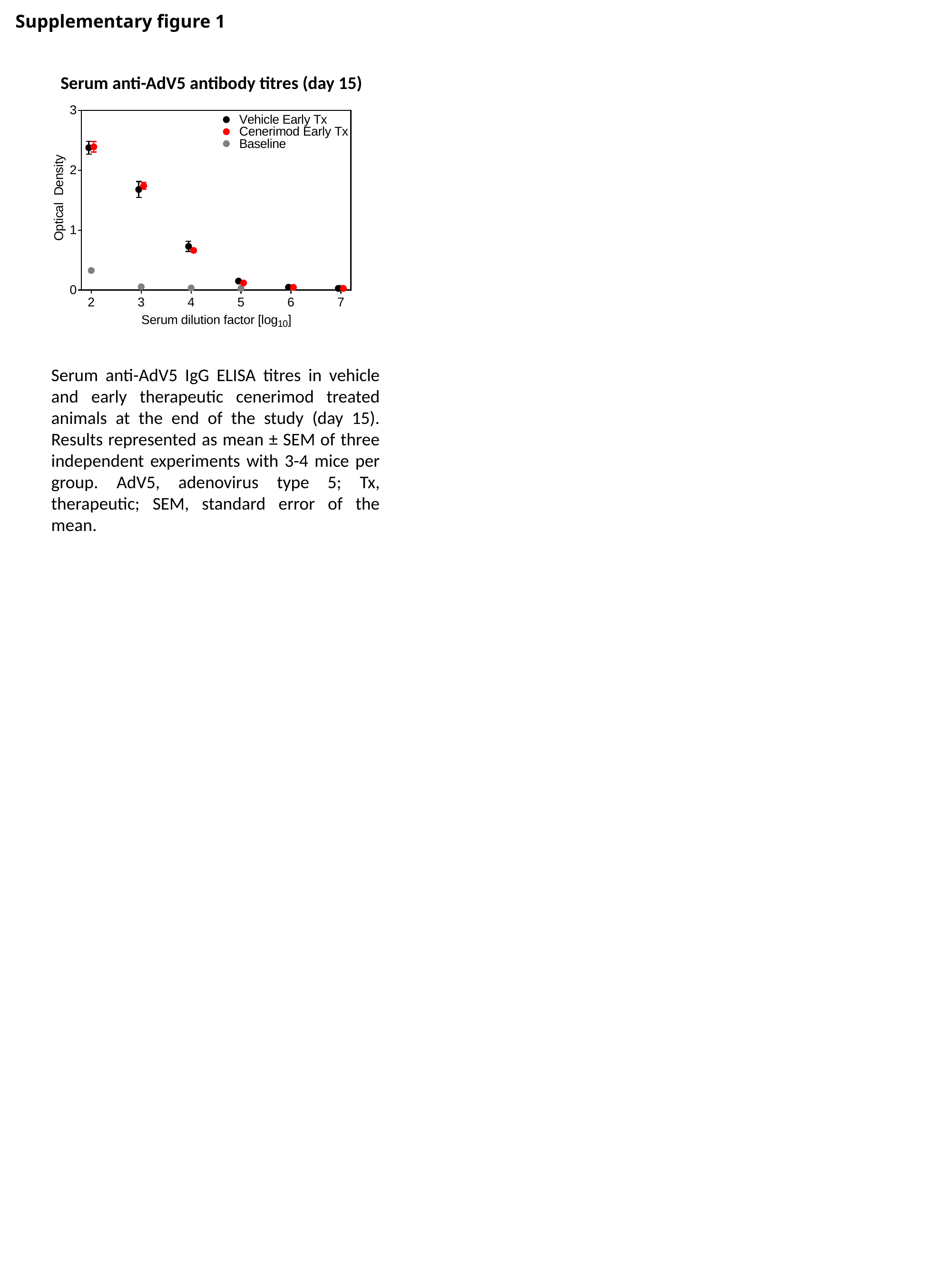

Supplementary figure 1
Serum anti-AdV5 antibody titres (day 15)
Serum anti-AdV5 IgG ELISA titres in vehicle and early therapeutic cenerimod treated animals at the end of the study (day 15). Results represented as mean ± SEM of three independent experiments with 3-4 mice per group. AdV5, adenovirus type 5; Tx, therapeutic; SEM, standard error of the mean.

## Slide 2
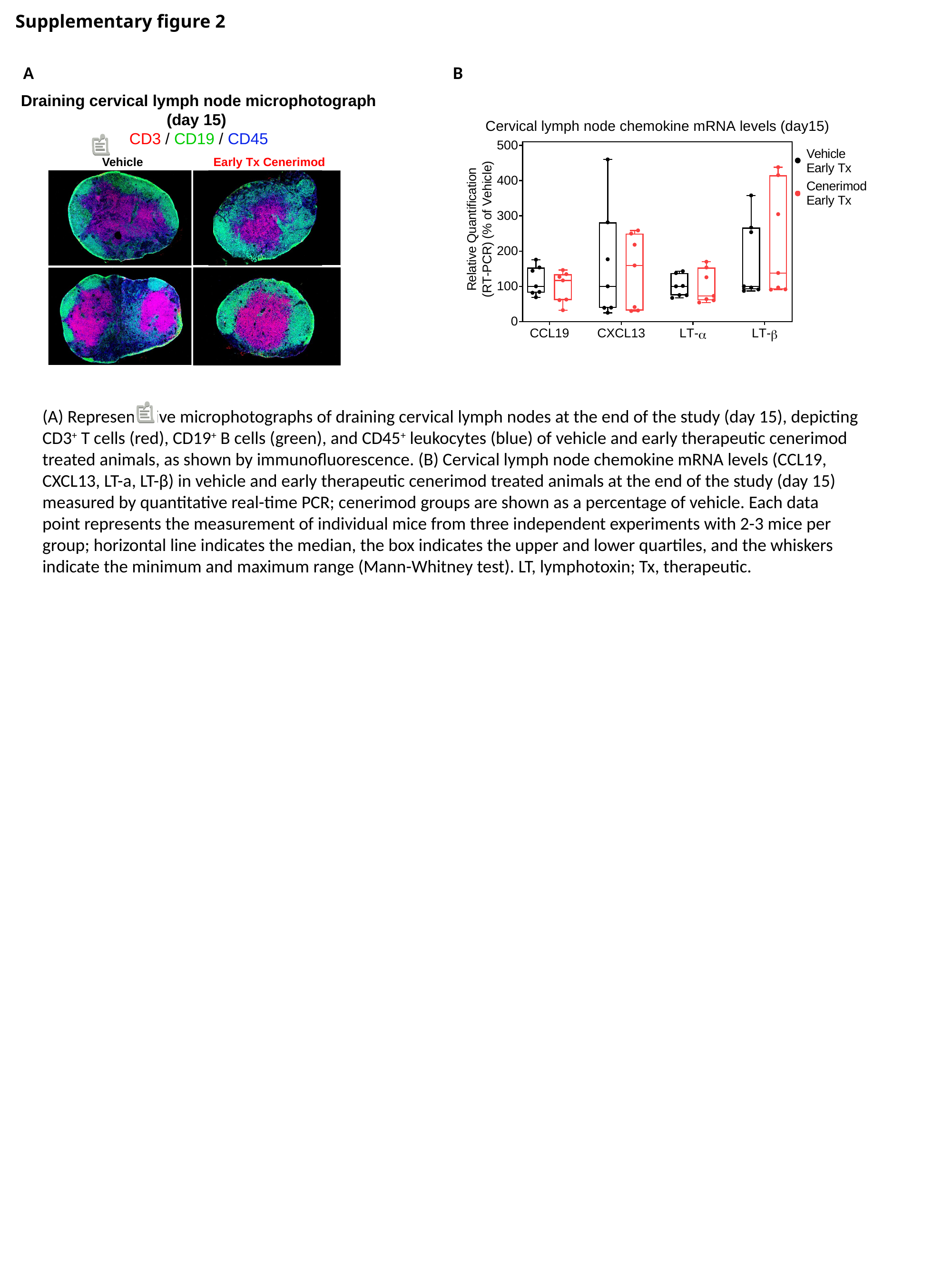

Supplementary figure 2
A
B
Draining cervical lymph node microphotograph
(day 15)
CD3 / CD19 / CD45
Vehicle
Early Tx Cenerimod
(A) Representative microphotographs of draining cervical lymph nodes at the end of the study (day 15), depicting CD3+ T cells (red), CD19+ B cells (green), and CD45+ leukocytes (blue) of vehicle and early therapeutic cenerimod treated animals, as shown by immunofluorescence. (B) Cervical lymph node chemokine mRNA levels (CCL19, CXCL13, LT-a, LT-β) in vehicle and early therapeutic cenerimod treated animals at the end of the study (day 15) measured by quantitative real-time PCR; cenerimod groups are shown as a percentage of vehicle. Each data point represents the measurement of individual mice from three independent experiments with 2-3 mice per group; horizontal line indicates the median, the box indicates the upper and lower quartiles, and the whiskers indicate the minimum and maximum range (Mann-Whitney test). LT, lymphotoxin; Tx, therapeutic.

## Slide 3
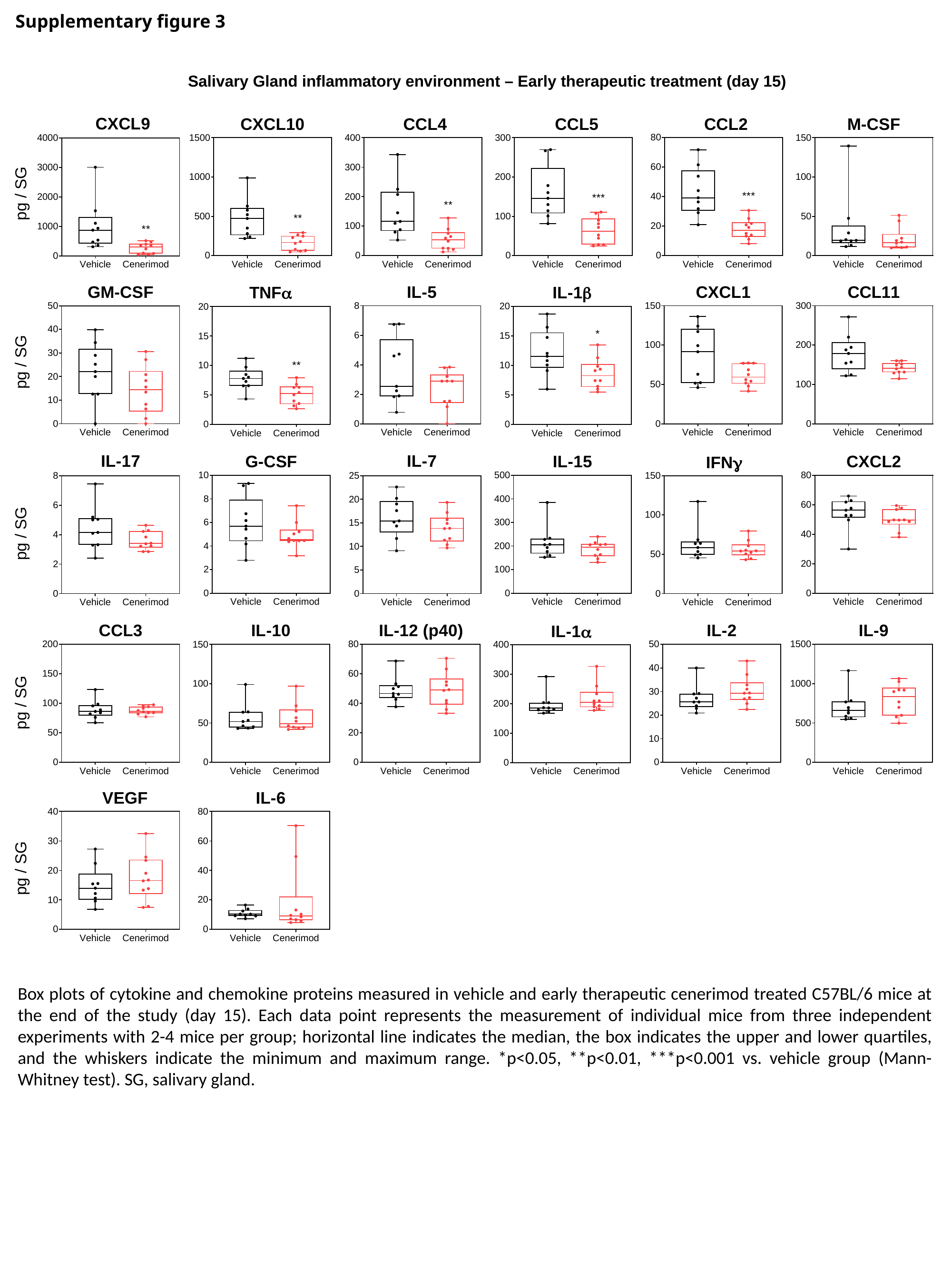

Supplementary figure 3
Salivary Gland inflammatory environment – Early therapeutic treatment (day 15)
pg / SG
pg / SG
pg / SG
pg / SG
pg / SG
Box plots of cytokine and chemokine proteins measured in vehicle and early therapeutic cenerimod treated C57BL/6 mice at the end of the study (day 15). Each data point represents the measurement of individual mice from three independent experiments with 2-4 mice per group; horizontal line indicates the median, the box indicates the upper and lower quartiles, and the whiskers indicate the minimum and maximum range. *p<0.05, **p<0.01, ***p<0.001 vs. vehicle group (Mann-Whitney test). SG, salivary gland.

## Slide 4
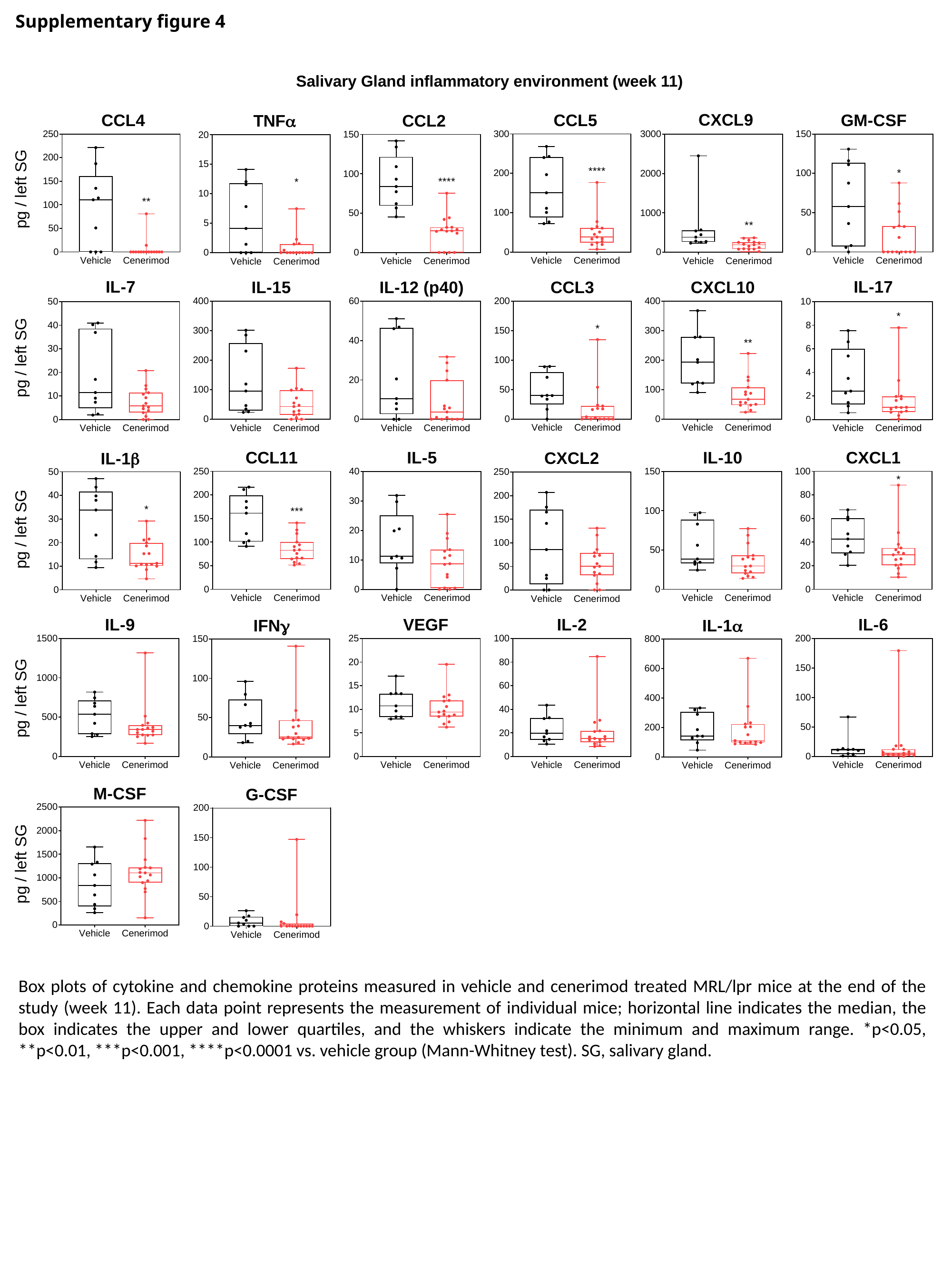

Supplementary figure 4
Salivary Gland inflammatory environment (week 11)
pg / left SG
pg / left SG
pg / left SG
pg / left SG
pg / left SG
Box plots of cytokine and chemokine proteins measured in vehicle and cenerimod treated MRL/lpr mice at the end of the study (week 11). Each data point represents the measurement of individual mice; horizontal line indicates the median, the box indicates the upper and lower quartiles, and the whiskers indicate the minimum and maximum range. *p<0.05, **p<0.01, ***p<0.001, ****p<0.0001 vs. vehicle group (Mann-Whitney test). SG, salivary gland.

## Slide 5
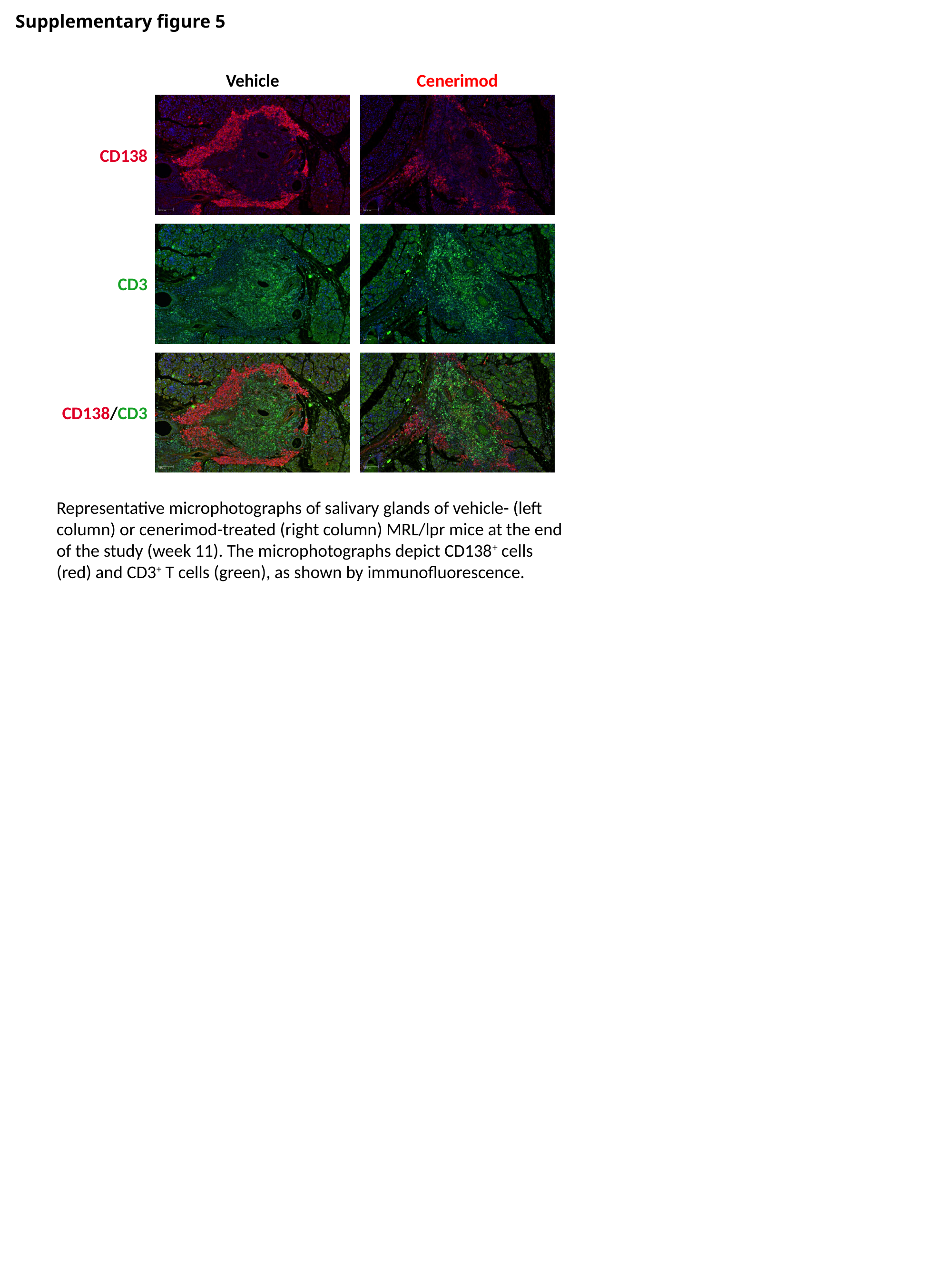

Supplementary figure 5
Vehicle
Cenerimod
CD138
CD3
CD138/CD3
Representative microphotographs of salivary glands of vehicle- (left column) or cenerimod-treated (right column) MRL/lpr mice at the end of the study (week 11). The microphotographs depict CD138+ cells (red) and CD3+ T cells (green), as shown by immunofluorescence.
